# Expression Analysis of Combinatorial Genes Using a Bi-Cistronic T2A Expression System in Porcine Fibroblasts

**DOI:** 10.1371/journal.pone.0070486

**Published:** 2013-07-29

**Authors:** Sunghoon Hurh, Bumrae Cho, Dong-Joo You, Hwajung Kim, Eun Mi Lee, Sang Hoon Lee, Sol Ji Park, Hayne Cho Park, Ok Jae Koo, Jaeseok Yang, Kook-Hwan Oh, Byeong Chun Lee, Jong-Ik Hwang, Curie Ahn

**Affiliations:** 1 Transplantation Research Institute, Seoul National University College of Medicine, Seoul, Republic of Korea; 2 Graduate School of Immunology, Seoul National University College of Medicine, Seoul, Republic of Korea; 3 Department of Theriogenology and Biotechnology, College of Veterinary Medicine, Seoul National University, Seoul, Republic of Korea; 4 Research Institute for Veterinary Science, Seoul National University, Seoul, Republic of Korea; 5 Graduate School of Medicine, Korea University, Seoul, Republic of Korea; 6 Department of Internal Medicine, Seoul National University College of Medicine, Seoul, Republic of Korea; 7 Institute of Green Bio Science & Technology, Seoul National University, Pyeongchang, Republic of Korea; 8 Transplantation Center, Seoul National University Hospital, Seoul, Republic of Korea; University of Valencia, Spain

## Abstract

In pig-to-primate xenotransplantation, multiple transgenic pigs are required to overcome a series of transplant rejections. The generation of multiple transgenic pigs either by breeding or the introduction of several mono-cistronic vectors has been hampered by the differential expression patterns of the target genes. To achieve simultaneous expression of multiple genes, a poly-cistronic expression system using the 2A peptide derived from the *Thosea asigna* virus (T2A) can be considered an alternative choice. Before applying T2A expression system to pig generation, the expression patterns of multiple genes in this system should be precisely evaluated. In this study, we constructed several bi-cistronic T2A expression vectors, which combine target genes that are frequently used in the xenotransplantation field, and introduced them into porcine fibroblasts. The proteins targeted to the same or different subcellular regions were efficiently expressed without affecting the localization or expression levels of the other protein. However, when a gene with low expression efficiency was inserted into the upstream region of the T2A sequences, the expression level of the downstream gene was significantly decreased compared with the expression efficiency without the insertion. A small interfering RNA targeting one gene in this system resulted in the significant downregulation of both the target gene and the other gene, indicating that multiple genes combined into a T2A expression vector can be considered as a single gene in terms of transcription and translation. In summary, the efficient expression of a downstream gene can be achieved if the expression of the upstream gene is efficient.

## Introduction

Due to the severe shortage of human donor tissues and organs, xenotransplantation has been considered as a potential alternative to allotransplantation. However, the clinical application of pig-to-primate xenotransplantation has been hampered by a series of obstacles, including hyperacute rejection (HAR), acute humoral xenograft rejection (AHXR), and cellular rejection [1]. Because of these complex and robust immune responses, it has become clear that the modulation of one or two target genes is insufficient for successful xenotransplantation. For example, the kidneys from α1,3-galactosyl transferase-knockout (GT-KO)/human CD46 (hCD46) transgenic pigs that were transplanted into baboons were rejected within 16 days [2], and the kidneys from GT-KO pigs transgenic for human CD55 (hCD55), hCD59, hCD39, and H-transferase (hHT) that were transplanted into baboons were rejected by AHXR within 15 days [3]. Thus, the generation of transgenic pigs that stably express multiple immune-modulating molecules is essential for overcoming xenograft rejection.

Multiple transgenic pigs have generally been produced by breeding [4] or via the transfection of multiple mono-cistronic plasmids containing target genes [5], [6]. However, breeding is time-consuming and expensive, and the target gene expression levels frequently decrease over time. Pigs generated by multiple plasmid transfections exhibit poorly synchronized expression of target genes. The alternative approach for generating multiple transgenic pigs is the use of a poly-cistronic expression system containing an internal ribosome entry site (IRES) or a viral 2A peptide. Various IRESs derived from viral genomes or eukaryotic messenger RNAs (mRNAs) have been widely distributed [7]. However, one of the major problems with the use of an IRES system is that the IRES-dependent expression of the second gene is significantly reduced compared with that of the first cap-dependent gene in mammalian cells [8], [9]. Furthermore, the translation efficiency was highly variable depending upon the origin of IRES or the transduced cell types [10], [11] because each IRES requires different IRES trans-acting factors (ITAFs) [12], [13].

Viral 2A peptides were initially identified in the viruses of the picornaviridae family, such as foot and mouth disease virus (FMDV) and cardiovirus. 2A peptides are composed of approximately 19 amino acids, including the consensus motif D(V/I)EXNPGP. “Self-cleaving” occurs through a ribosomal skipping mechanism, which might inhibit the formation of a peptide bond between the glycine and proline residues within the consensus motif [14]. When a 2A peptide exists between two genes, after the translation of the upstream gene, the ribosome skips translation at the glycine-proline junction in the 2A peptide and continues to translate the downstream gene. Previous studies have shown that the efficiency of this ribosomal skip is highly variable, depending on the sequences in upstream region of the consensus motif in the 2A peptides [15]. The efficiencies of the ribosomal skip were not equal among representative 2A peptides [16], such as the F2A peptide from FMDV, E2A from the equine rhinitis A virus, P2A from the porcine teschovirus-1, and T2A from the *Thosea asigna* virus [17]–[19]. Therefore, the selection of an optimal 2A peptide is determinant for the stable expression of target genes. Donnelly et al. previously tested several 2A peptides from different viral genes and showed that the T2A peptide exhibited favorable cleavage efficiency [15]. For this reason, we used the T2A peptide as a linker for all of the bi-cistronic vectors used in this study.

Although 2A peptides are useful for the simultaneous expression of multiple genes at the same site and have received significant attention in the xenotransplantation field, factors influencing the expression levels of the target genes in a poly-cistronic T2A expression (T2A-Ex) system have not been fully elucidated. Several studies have shown that 2A peptide-mediated gene expression might be influenced by protein cleavage and the positioning of the genes within the vector [20]–[22]. For example, although most proteins are synthesized in ribosomal complexes, some proteins that localize to the plasma membrane are also integrated within the endoplasmic reticulum (ER) but not in the cytosolic fractions. Therefore, differential subcellular localization might influence the expression pattern of target genes coupled to the 2A peptide.

Because the stable and consistent expression of all of the target genes is essential for xenograft survival, we evaluated the impact of the gene positioning and subcellular localization of target genes on their expression patterns using bi-cistronic T2A-Ex constructs driven by a CMV promoter. The combination of four commonly used genes, including the enhanced green fluorescent protein (EGFP), HA-tagged human heme oxygenase 1 ((HA)HO1), human thrombomodulin (hTBM), and hCD46, were used to determine the protein expression patterns using the T2A-Ex system.

## Materials and Methods

### Cells and Cell Culture

Porcine fibroblasts were isolated from White Yucatan miniature pig fetuses on day 35 of gestation as previously described [23]. The cells were kindly provided by Dr. Hyunil Kim and OPTIFARM SOLUTION. The cells were maintained in Dulbecco’s modified Eagle medium (DMEM; WelGENE, Daegu, Korea) supplemented with 20% (v/v) fetal bovine serum (FBS; Gibco, MD, USA) and 1% (v/v) antibiotic-antimycotic solution (Gibco, MD, USA) at 38°C in a humidified carbon dioxide-controlled (5%) incubator.

### Plasmid Construction and Transfection

The bi-cistronic gene combinations used in this study are summarized in Table 1 and the primers used in this study are summarized in Table S1. For the IRES-related constructs, each upstream gene was inserted into the pIRES vector (Clontech, CA, USA) using *Nhe*I and *Xho*I. After the insertion of the upstream gene, each downstream gene was inserted using *Bam*HI and *Not*I. In the T2A-Ex constructs, each of the upstream genes except for hTBM was inserted into the pBlue-T2A vector (pBluescript II KS(-) vector including a T2A peptide with an N-terminal furin cleavage sequence at the *Eco*RV site) using *Kpn*I and *Eco*RI. The hTBM gene was inserted into the pBlue-T2A vector between the *Bam*HI and *Eco*RI. Each of downstream genes was inserted using *Hin*dIII and *Xho*I. After the insertion of both upstream and downstream genes, the entire coding region was subcloned into the pcDNA3.1(+) or pCAG1.1 vectors (in which the CMV promoter was exchanged with the CAG promoter in pcDNA3.1(+) vector) using *Kpn*I and *Xho*I, or *Bam*HI and *Xho*I, respectively. The stop codons of all upstream genes were deleted in the bi-cistronic T2A-Ex vector. After construction of the bi-cistronic T2A-Ex vectors, EGFP-T2A-(HA)HO1 and hTBM-T2A-(HA)HO1 sequences were used for construction of the tri-cistronic T2A-Ex vectors. Upstream EGFP-T2A-(HA)HO1 and hTBM-T2A-(HA)HO1 sequences without stop codon were amplified by polymerase chain reaction (PCR) and 5′-regions were digested by *Kpn*I or *Bam*HI, respectively. Downstream T2A-hCD46 sequences were amplified and 3′-region was digested by *Xho*I. After treatment with T4 poly nucleotide kinase (Elpis Biotech, Daejeon, Korea), upstream and downstream sequences were inserted together into pcDNA3.1(+) using *Kpn*I and *Xho*I or *Bam*HI and *Xho*I.

**Table 1:** The combinations of target genes within the bi-cistronic T2A-Ex vector.

Promoter	Gene A	Gene B	Verification
CMV^1^	(HA)EGFP^2^	(Myc)EGFP	Position
	(Myc)EGFP	(HA)EGFP	
	EGFP	(HA)HO1^3^	Intracellular protein
	(HA)HO1	EGFP	
	hTBM^4^	hCD46^5^	Transmembrane protein
	hCD46	hTBM	
	EGFP	hTBM	Mixed
	hTBM	EGFP	
	hCD46	EGFP	–
	EGFP	hCD46	
	hTBM	(HA)HO1	

1 Cytomegalovirus.

2 Enhanced green fluorescent protein.

3 Human heme oxygenase 1.

4 Human thrombomodulin.

5 Human cluster of differentiation 46.

To examine the protein expression pattern of these constructs, 2 or 3 µg of each plasmid were transiently introduced into 1×10^6^ porcine fibroblasts. For RNA interference, we used 1 µg of small interfering RNA (siRNA) targeting GFP or luciferase GL2 (siGFP or siLuc, respectively; GenePharma, Shanghai, China). The sequences of the siRNAs were designated as follows: siGFP, 5′-GGCUACGUCCAGGAGCGCACC-3′ and 5′-UGCGCUCCUGGACGUAGCCUU-3′, and siLuc, 5′-CGUACGCGGAAUACUUCGAdTdT-3′ and 5′-UCGAAGUAUUCCGCGUACGdTdT-3′. For transfection, we electroporated the cells using Nucleofector II™ and the Nucleofector™ Kit V (Lonza, Cologne, Germany) according to the manufacturer’s protocols.

### Flow Cytometry

Detached cells were incubated in phosphate-buffered saline (PBS; Invitrogen, CA, USA) with 2% (w/v) bovine serum albumin (BSA; Invitrogen, CA, USA) and the indicated antibodies. For intracellular staining, FIX & PERM™ and permeabilization buffers (Invitrogen, CA, USA) were used. For the detection of the HA-epitopes, a mouse anti-HA-Tag antibody (1∶200; Abcam, MA, USA) and allophycocyanin (APC)-conjugated goat anti-mouse IgG antibody (1∶50; Santa Cruz Biotechnology, CA, USA) were used sequentially. For the detection of hTBM and hCD46, APC-conjugated mouse anti-human TBM (1∶100; R&D Systems, MN, USA) and CD46 (1∶100; Abcam, MA, USA) antibodies were used, respectively. The immunostained cells were analyzed using a FACSCalibur flow cytometer (Becton Dickinson, CA, USA) with FlowJo software (Tree Star, OR, USA). The levels of the proteins expressed by multiple gene constructs were determined more than three times each and applied for quantitative analysis, as shown in Table 2.

**Table 2:** The quantitative analysis of each gene by multi-cistronic vectors used in this study.

				MFI (Mean±SE)			
Promoter	Gene combination	Linker	N	EGFP	(HA)HO1	hTBM	hCD46
CMV	EGFP–(HA)HO1	IRES	3	690.0±95.7	31.9±6.7		
	(HA)HO1–EGFP	IRES	3	21.1±3.9	95.5±19.7		
	EGFP–(HA)HO1	T2A	8	755.6±74.0	129.6±11.4		
	(HA)HO1–EGFP	T2A	7	306.3±48.8	109.0±13.3		
	hTBM–hCD46	T2A	7			584.5±23.8	690.0±24.8
	hCD46–hTBM	T2A	3			53.6±5.2	96.5±7.6
	EGFP–hTBM	T2A	4	752.6±135.0		840.2±73.0	
	hTBM–EGFP	T2A	7	79.2±4.3		563.9±11.2	
	hCD46–EGFP	T2A	3	13.1±2.9			85.4±12.8
	EGFP–hCD46	T2A	4	742.1±108.9			1201.4±50.4
	hTBM–(HA)HO1	T2A	4		69.8±15.7	558.9±9.8	
	EGFP–(HA)HO1–hCD46	T2A	4	729.7±63.9	125.8±16.1		1128.1±84.2
	hTBM–(HA)HO1–hCD46	T2A	4		64.3±14.7	516.5±21.2	627.1±25.7

### Western Blotting

The transfected cells were harvested and lysed in RIPA buffer (BIOSESANG, Seongnam, Korea) supplemented with a protease inhibitor cocktail (Complete Mini; Roche, NJ, USA). Five micrograms of each cell lysate were quantified using a Bradford assay reagent (BIOSESANG, Korea) and loaded per lane. The lysate samples were resolved using SDS-PAGE and transferred to PVDF membranes (Merck Millipore, MA, USA). The membranes were blocked in TBS containing 0.1% (v/v) Tween-20 (TBST; Bio-Rad, CA, USA) and 5% (w/v) skim milk (Becton Dickinson, CA, USA). After 1 hour of blocking at room temperature, the membranes were sequentially incubated with the indicated primary and secondary antibodies diluted in TBST containing 2% (w/v) BSA (Invitrogen, CA, USA). The following primary antibodies were used for immunoblotting: mouse anti-GFP antibody (1∶5000; Santa Cruz Biotechnology, CA, USA), mouse anti-HA-Tag antibody (1∶4000; Abcam, MA, USA), mouse anti-Myc-Tag antibody (1∶2000; Cell Signaling, MA, USA), mouse anti-hTBM antibody (1∶1000; Abcam, MA, USA), and rabbit anti-hCD46 antibody (1∶1000; Abcam, MA, USA). The following secondary antibodies were used for immunoblotting: horseradish peroxidase (HRP)-conjugated goat anti-mouse IgG (1∶10000; AbFrontier, Seoul, Korea) and HRP-conjugated goat anti-rabbit IgG (1∶5000; AbFrontier, Seoul, Korea). After three washes with TBST, chemiluminescent detection was performed using the AbSignal™ Kit (AbClon, Seoul, Korea), followed by X-ray film exposure.

### Fluorescent Microscopy

Coverslips were incubated with 0.01% (w/v) poly-L-lysine solution (Sigma, MO, USA) for 1 hour. After washing with 70% ethanol, the coverslips were placed into the individual wells of a six-well plate. Ten thousand transfected cells were seeded into each well. After washing twice with 1X PBS, the cells were fixed with 3.7% (w/v) paraformaldehyde at room temperature for 30 minutes. After washing with 1X PBS, the cells were incubated with 2 µg/ml of Hoechst 33342 at room temperature for 10 minutes to stain the nuclei (DNA) of the cells. After three washes with 1X PBS, GFP expression and Hoechst 33342 were examined using a Zeiss LSM 410 confocal microscope (Carl Zeiss AG, Oberkochen, Germany).

### RNA Isolation and Semi-quantitative Reverse Transcription Polymerase Chain Reaction

Total RNA was isolated from the transfected cells using the RNeasy™ Mini Kit (Qiagen, CA, USA) according to the manufacturer’s protocols. To remove the residual plasmid DNA, 1 mg of total RNA was incubated with 0.2 units of RQ1 RNase-Free DNase (Promega, WI, USA) at 37°C for 30 minutes. After the addition of 0.2 µl of RQ1 DNase Stop Solution, the samples were incubated at 65°C for 10 minutes to inactivate the DNase. After the removal of the residual plasmid DNA, the total RNA was reverse transcribed into complementary DNA (cDNA) using SuperScript™ III Reverse Transcriptase (Invitrogen, CA, USA) according to the manufacturer’s protocols. Next, 23 cycles of polymerase chain reaction (PCR) were performed using a PCR Thermal Cycler Dice™ (TAKARA, Shiga, Japan) and HiPi Thermostable DNA Polymerase (Elpis Biotech, Daejeon, Korea) with the EGFP or β-Actin primer pairs summarized in Table S1. The PCR products were electrophoresed on 1.5% (w/v) agarose gels in 0.5X TBE buffer and visualized by UV transillumination.

### Statistical Analysis

The SPSS version 18.0 (SPSS Inc., IL, USA) was used for statistical analysis. Unpaired Student’s *t*-test was used to compare results between groups. *P*-values less than 0.05 were considered statistically significant (*, *p*<0.05; **, *p*<0.01; ***, *p*<0.001; ns, not significant). Error bars represent standard error of the mean.

## Results

### IRES-dependent Downstream Genes are not Efficiently Expressed

**Figure 1:**
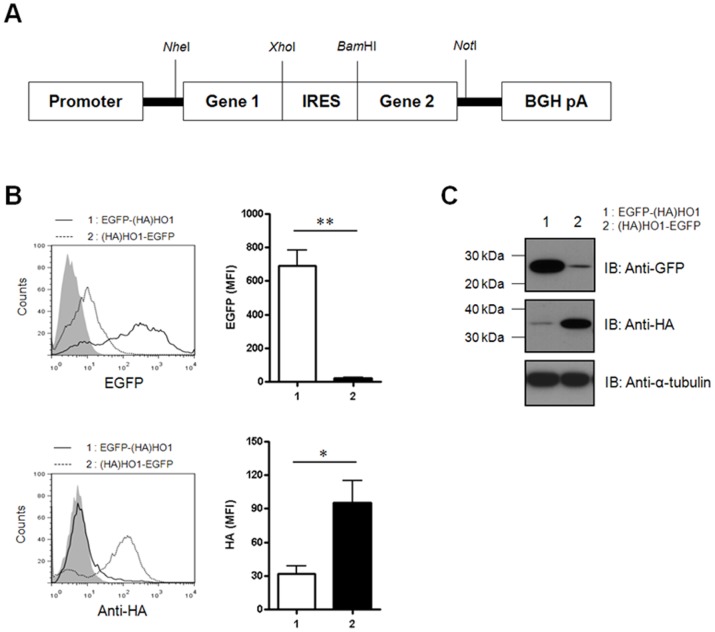
The expression levels of downstream genes are extremely low in the bi-cistronic IRES-Ex constructs. **A:** Schematic diagram of the bi-cistronic IRES-Ex vector. **B:** Porcine fibroblasts were transfected with the vector containing the EGFP-IRES-(HA)HO1 or (HA)HO1-IRES-EGFP sequences. The cells were analyzed using flow cytometry at 24 h after transfection. For (HA)HO1 detection, the cells were incubated with the mouse anti-HA Ab, followed by the APC-conjugated goat anti-mouse IgG Ab, according to the intracellular staining protocol. Mock transfected cells were used as control (filled line). Summarized bar graphs show means ± SE of three independent replications. **C:** The cell lysates were subjected to immunoblotting with either the mouse anti-GFP or mouse anti-HA Abs, followed by HRP-conjugated goat anti-mouse IgG. These results are representative of three independent experiments.

It has been well established that the IRES sequence does not produce efficient gene expression in mammalian cells [8], [9]. To resolve this issue, we used the bi-cistronic IRES expression (IRES-Ex) system shown in Figure 1A. We constructed two bi-cistronic IRES-Ex vectors containing upstream and downstream combinations of the EGFP and (HA)HO1 genes and introduced each construct into porcine fibroblasts. Using flow cytometric analysis, we found that the expression levels of the downstream EGFP and (HA)HO1 genes were significantly reduced compared with those of the upstream EGFP and (HA)HO1 genes (Figure 1B). The expression levels of both genes were also confirmed by immunoblot analysis using the antibodies specific for each gene. Consistent with the flow cytometric results, the expression of the downstream EGFP and (HA)HO1 was barely detectable (Figure 1C).

### The Position of the Target Gene within the T2A-Ex Vector does not Affect its Expression Levels

**Figure 2:**
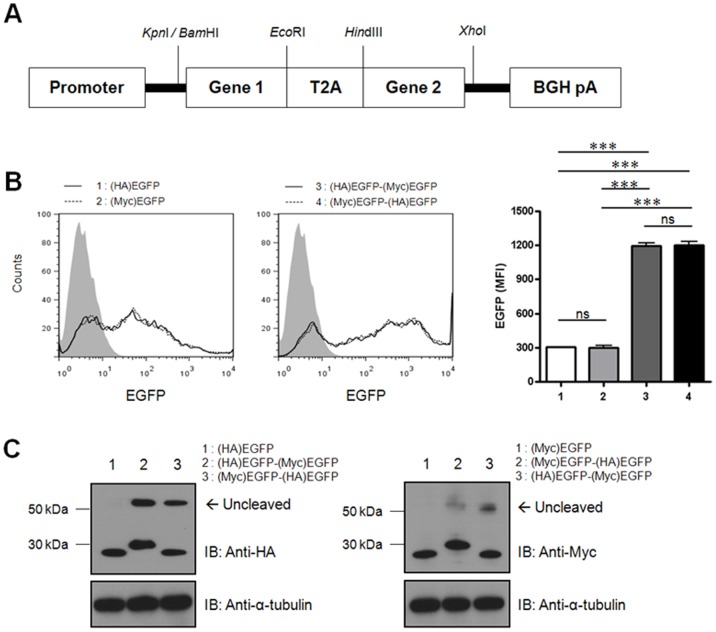
The expression levels of downstream genes are approximately equivalent to those of upstream genes. **A:** Schematic diagram of bi-cistronic T2A-Ex. **B:** Porcine fibroblasts were transfected with vector containing a single gene, (HA)EGFP or (Myc)EGFP, or vector containing a combination of genes, (HA)EGFP-T2A-(Myc)EGFP or (Myc)EGFP-T2A-(HA)EGFP. At 24 h after transfection, the EGFP signals were analyzed by flow cytometry. Mock transfected cells were used as control (filled line). Summarized bar graphs show means ± SE of three independent replications. **C:** The cell lysates were examined by immunoblot analysis with the mouse anti-HA or mouse anti-Myc Abs, followed by the HRP-conjugated goat anti-mouse IgG. Two micrograms of each plasmid were used in this figure. These results are representative of three independent experiments.

A schematic diagram of the bi-cistronic T2A-Ex constructs is presented in Figure 2A. To compare the expression levels of the upstream genes with those of downstream genes, either HA- or Myc-tagged EGFP ((HA)EGFP or (Myc)EGFP) were used. First, we constructed two mono-cistronic vectors containing either the (HA)EGFP or (Myc)EGFP sequences and confirmed similar levels of EGFP expression by flow cytometry. Next, bi-cistronic vectors containing (HA)EGFP-T2A-(Myc)EGFP or (Myc)EGFP-T2A-(HA)EGFP sequences were generated. Flow cytometric analysis using porcine fibroblasts transfected with each bi-cistronic vector revealed that both constructs produced similar EGFP signals. However, these signals were stronger than those of the mono-cistronic vectors, suggesting that two copies of the EGFP gene in a single transcript resulted in increased protein synthesis compared to one copy (Figure 2B). The expression levels of both upstream and downstream EGFP genes were further confirmed by immunoblot analysis. The data showed that the expression levels of the downstream EGFP were similar to those of the upstream EGFP and the EGFP derived from mono-cistronic vector. Tagging using different epitopes did not affect EGFP expression levels (Figure 2C).

### The Combination of Genes Targeted for Different Subcellular Regions does not Influence the Protein Expression Efficiency in the T2A-Ex System

To explore whether the targeting of genes to different subcellular locations can influence the expression patterns of multigene, we generated six bi-cistronic T2A-Ex vectors containing combinations of two intracellular proteins, EGFP and (HA)HO1, and two trans-membrane proteins, hTBM and hCD46.

**Figure 3:**
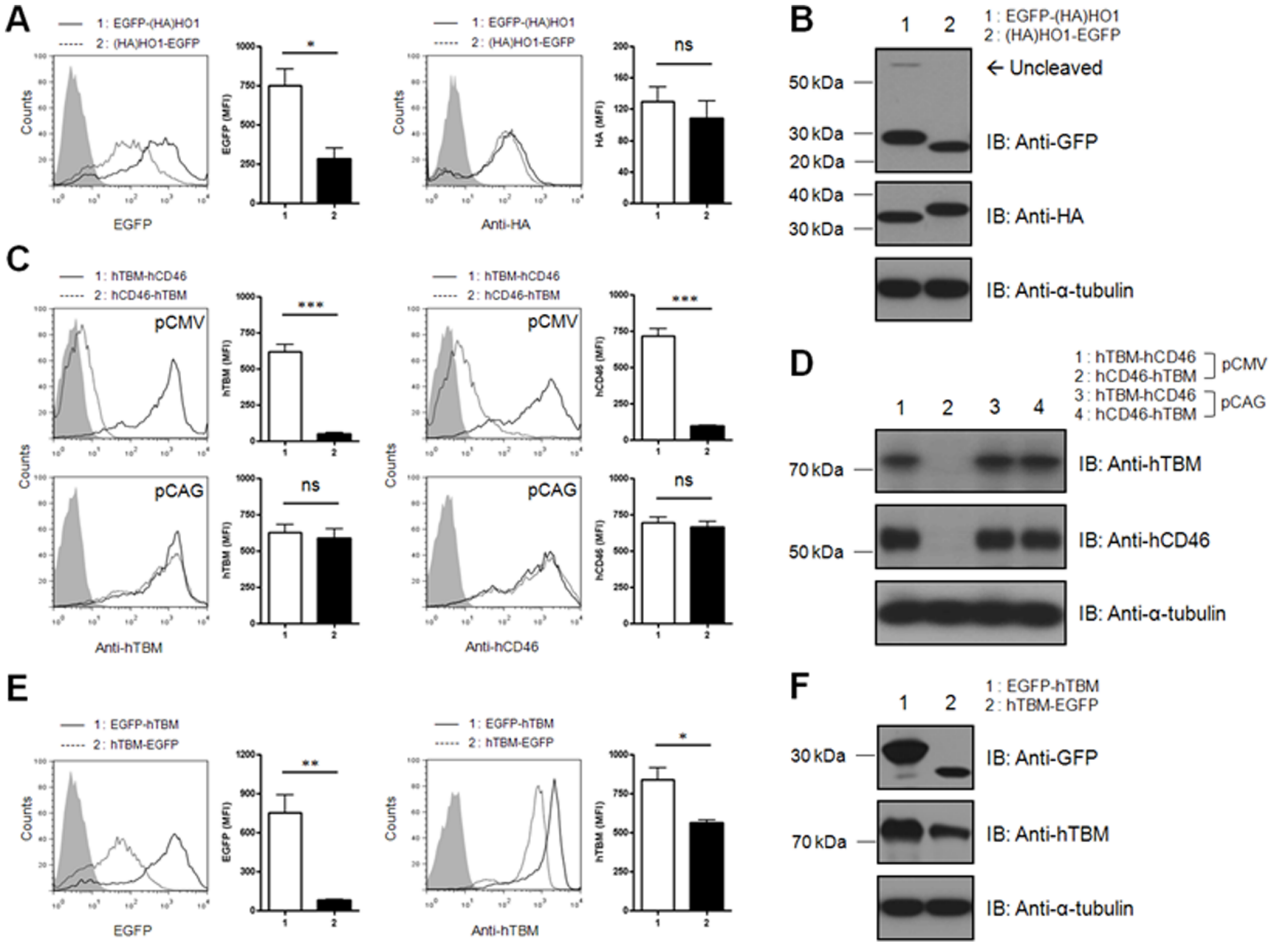
Both genes in the bi-cistronic T2A-Ex constructs are localized efficiently to their subcellular location. **A–B:** The bi-cistronic T2A-Ex vector containing the EGFP-T2A-(HA)HO1 or (HA)HO1-T2A-EGFP sequences was introduced into porcine fibroblasts, and the expression patterns of each gene were analyzed by flow cytometry (A) and western blotting (B) with the indicated antibodies. Mock transfected cells were used as control (filled line). Summarized bar graphs show means ± SE of four independent replications. **C:** Porcine fibroblasts transfected with CMV- (upper panel) or CAG-based (lower panel) bi-cistronic T2A-Ex vector containing the hTBM-T2A-hCD46 or hCD46-T2A-hTBM sequences. The cell surface expression of hTBM or hCD46 was analyzed by flow cytometry using the APC-conjugated mouse anti-hTBM or APC-conjugated mouse anti-hCD46 antibodies, respectively. Mock transfected cells were used as control (filled line). Summarized bar graphs show means ± SE of three independent replications. **D:** The cell lysates were subjected to immunoblotting with the mouse anti-hTBM and rabbit anti-hCD46 antibodies, followed by the HRP-conjugated goat anti-mouse IgG or HRP-conjugated goat anti-rabbit IgG, respectively. **E–F:** The expression patterns of target genes in the porcine fibroblasts transfected with the bi-cistronic T2A-Ex vector containing the EGFP-T2A-hTBM or hTBM-T2A-EGFP sequences were analyzed by flow cytometry (E) and western blotting (F) with the indicated antibodies. Mock transfected cells were used as control (filled line). Summarized bar graphs show means ± SE of four independent replications. These results are representative of more than three independent experiments.

Immunostaining with an anti-HA antibody, followed by flow cytometric analysis, showed that (HA)HO1 was expressed relatively efficiently, regardless of its position within the bi-cistronic T2A-Ex construct. The EGFP signals were rather different according its sequence position. When the EGFP gene was located within the downstream region, its expression levels were quite reduced compared with those of the EGFP gene within the upstream region (Figure 3A). However, compared with the bi-cistronic IRES-Ex construct (Figure 1B and 1C), the bi-cistronic T2A-Ex construct maintained significantly high expression of each gene within the downstream position (EGFP, *p* = 0.022; (HA)HO1, *p* = 0.009; data not shown). These results were confirmed by immunoblot analysis. Both the EGFP and (HA)HO1 proteins were clearly detectable, regardless of their position within the bi-cistronic T2A-Ex construct, even though the signal of the downstream EGFP was negligibly weaker than that of the upstream EGFP. The molecular sizes of the upstream genes were slightly larger due to the addition of a C-terminal T2A peptide, indicating that furin-dependent cleavage did not occur in the cytosol, although the protease target site was inserted upstream of T2A peptide (Figure 3B).

The expression pattern of the two trans-membrane molecules, hTBM and hCD46, were examined using the bi-cistronic T2A-Ex constructs containing the hTBM-T2A-hCD46 or hCD46-T2A-hTBM sequences, respectively. The porcine fibroblasts transfected with the hTBM-T2A-hCD46 vector expressed both hTBM and hCD46 proteins at the cell surface. However, in flow cytometric analysis with the hCD46-T2A-hTBM-transfected cells, the protein expression levels of both hCD46 and hTBM were extremely low compared with hTBM-T2A-hCD46-transfected cells. Similar to this, they were undetectable in western blotting (Figure 3C and 3D).

To evaluate the expression of the combined genes in the context of different subcellular localization, bi-cistronic T2A-Ex constructs containing either EGFP-T2A-hTBM or hTBM-T2A-EGFP sequences were generated. Flow cytometric analysis showed that both EGFP and hTBM proteins were well expressed at their differential subcellular locations. Interestingly, however, the protein expression levels of both genes were quite different between the groups (Figure 3E). Immunoblot analysis showed that the upstream EGFP was present at an increased size due to the addition of the T2A peptide. However, the upstream hTBM was detected at its original size due to cleavage by furin, which is present in the ER lumen (Figure 3F).

### The Influence of Upstream Gene Expression on Downstream Gene Expression in the T2A-Ex Constructs

In Figure 3A, reduced EGFP expression was observed when the EGFP gene was located within the downstream region. The expression of the EGFP protein was also decreased when the EGFP gene was located within the downstream region of the hTBM gene (Figure 3E). Moreover, in hCD46-T2A-hTBM-transfected cells, negligible expression of hTBM protein was observed, although when the hTBM gene was located in the upstream position or in the mono-cistronic vector, it was highly expressed (Figure 3C and data not shown). Interestingly, hCD46 was expressed efficiently by the T2A-Ex construct containing hTBM-T2A-hCD46, whereas when the hCD46 gene was located in the upstream position or in the mono-cistronic vector, it was not efficiently expressed. In this study, we used a CMV promoter-derived gene expression system. The other genes were expressed efficiently by this promoter. However, the hCD46 gene was not expressed efficiently. To determine the promoter dependency of the hCD46 gene, we subcloned hCD46-T2A-hTBM sequences into a chicken β-actin (CAG) promoter-containing vector. Because the upstream hCD46 gene was expressed efficiently under the control of the CAG promoter, the downstream hTBM gene was also expressed efficiently. The protein expression levels of both genes were not different from hTBM-T2A-hCD46-transfected group in CAG promoter-driven expression system (Figure 3C and 3D). These findings suggest that the expression efficiency of the upstream genes may influence the expression efficiency of the downstream genes.

**Figure 4:**
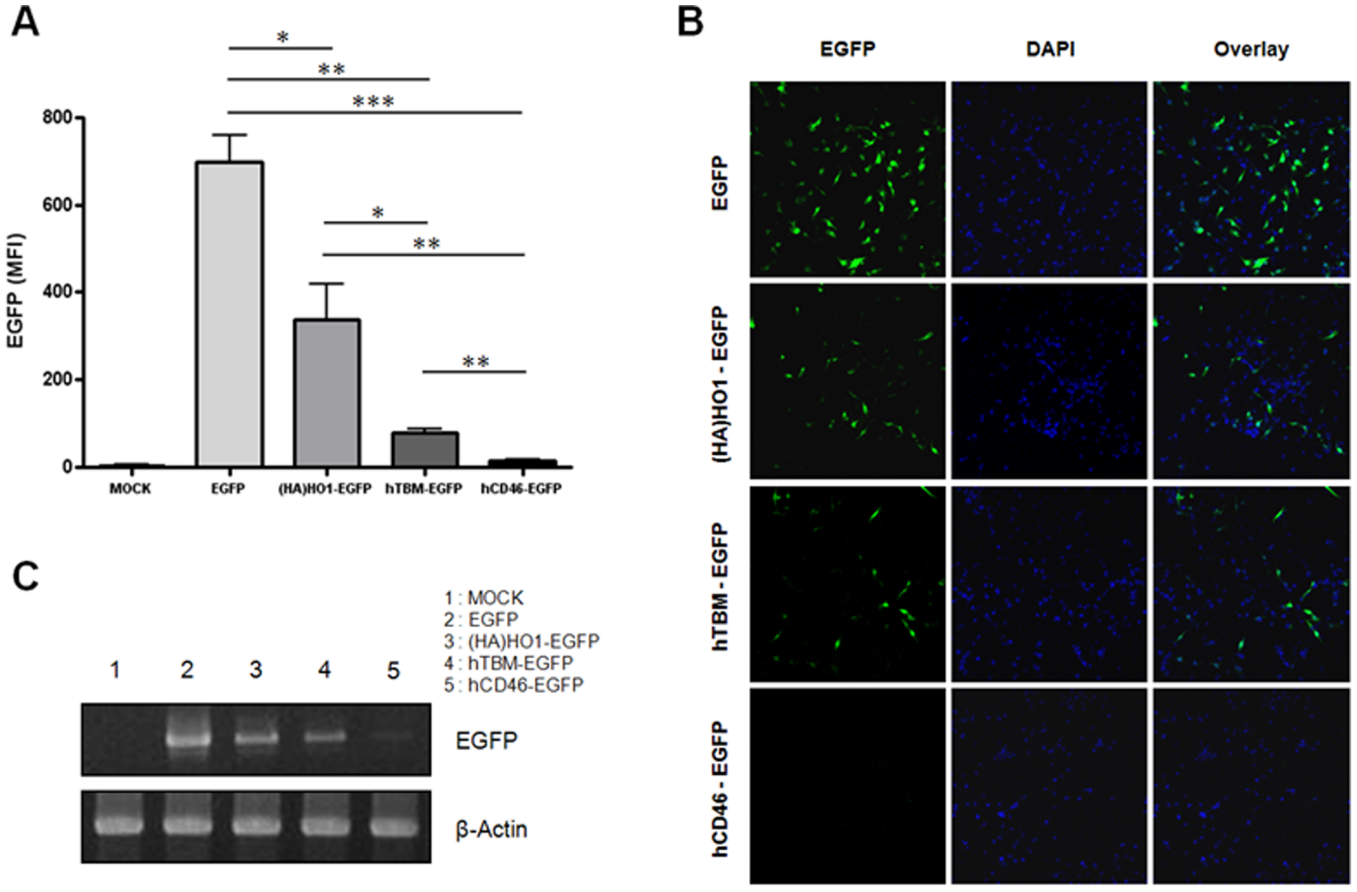
The downstream gene expression levels are correlated with the transcriptional efficiency of the upstream gene. **A:** Porcine fibroblasts were transfected with each indicated vector, and the mean fluorescent intensity (MFI) of EGFP was analyzed by flow cytometry. Summarized bar graphs show means ± SE of three independent replications. **B:** The expression levels and the subcellular localization of EGFP were analyzed using a fluorescent microscope. Hoechst 33342 was used for nuclear staining (original magnification X200). **C:** After the removal of the residual plasmid DNA with DNase treatment, the RNA extracts were subjected to RT-PCR for EGFP. These results are representative of three independent experiments.

To exclude the interference of different antibody binding affinities, we generated a structural sequence of Gene A–T2A–EGFP. After inserting the three different genes ((HA)HO1, hTBM, and hCD46) into the Gene A position, each bi-cistronic vector was introduced into porcine fibroblasts, and the mean fluorescent intensity (MFI) of EGFP was measured by flow cytometry. The EGFP expression levels decreased in order of combination with (HA)HO1, hTBM, and hCD46 (Figure 4A). The EGFP signals from each construct observed by fluorescence microscopy were correlated with the flow cytometry results (Figure 4B). To identify whether different protein expression levels of the downstream EGFP gene were correlated with the mRNA expression levels, we assessed the mRNA levels using reverse transcription polymerase chain reaction (RT-PCR) for EGFP because all of the constructs contained EGFP sequences at the same position. The data showed that different amounts of the mRNA existed in each group and that the mRNA levels were significantly correlated with the protein levels (Figure 4C). The different mRNA levels might be due to the transcriptional efficiency of each construct or the stability of the transcripts.

**Figure 5:**
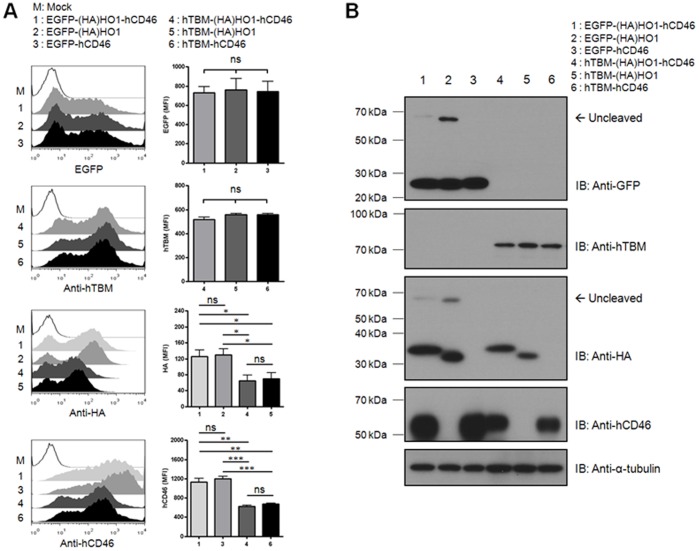
The expression levels of each gene are not related with differences in subcellular localization. **A–B:** The bi- and tri-cistronic T2A-Ex vectors containing the indicated gene combinations were introduced into porcine fibroblasts. The expression levels of each gene were analyzed by flow cytometry (A) and western blotting (B) with the indicated antibodies. Summarized bar graphs show means ± SE of four independent replications. These results are representative of four independent experiments.

To determine whether the expression levels of the downstream genes were influenced by those of upstream genes in the context of subcellular localization, EGFP and hTMB were selected as upstream genes because EGFP expressed much more efficiently than hTBM in our system. (HA)HO1 and hCD46 were used as the downstream genes. The expression levels of EGFP and hTBM were consistent and not influenced by the downstream genes. However, (HA)HO1 and hCD46 were expressed much more efficiently when EGFP was positioned within the upstream region compared with hTMB (Figure 5A and 5B). To investigate if expression efficiency of the first gene influences downstream gene expression in triple gene construct, we developed tri-cistronic T2A-Ex system with combination of EGFP-T2A-(HA)HO1-T2A-hCD46 or hTBM-T2A-(HA)HO1-T2A-hCD46. Flow cytometric analysis and western blotting data showed that expression levels of hCD46 in the third position were also related to the expression efficiency of the first gene (EGFP or hTBM). Furthermore, the expression levels of hCD46 in the third position were quite similar to those of hCD46 in second position of bi-cistronic construct (Figure 5A and 5B).

These results indicate that the expression level of downstream genes appears to be dependent on the transcriptional efficiency of upstream genes in the T2A-Ex constructs, regardless of their targeted subcellular localization.

### Multigene Expression Driven by a Single Promoter can be Regulated by RNA Interference of One Gene Member

Small RNAs can bind to specific regions of gene transcripts with which they share significant homology, resulting in the degradation or translational disruption of the transcripts, a phenomenon called RNA interference [24], [25]. Because the poly-cistronic T2A-Ex construct produces a single transcript containing multiple genes, a microRNA (miRNA) targeting one gene within the transcript would simultaneously downregulate the expression of the other genes.

**Figure 6:**
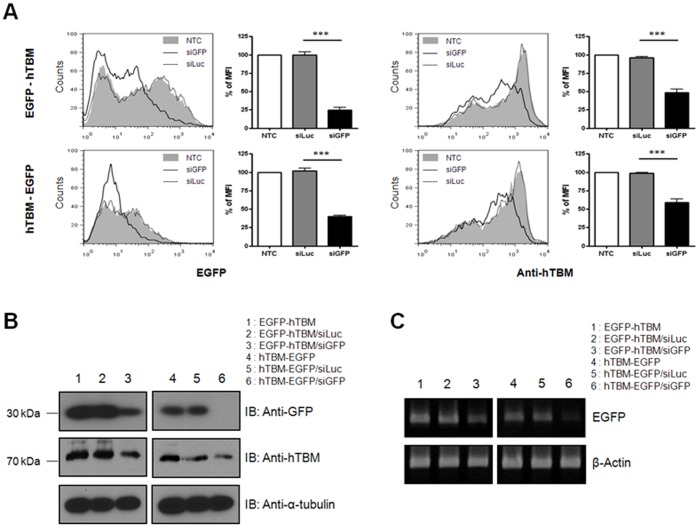
The siRNA targeting of one gene can downregulate both genes in bi-cistronic T2A-Ex constructs. **A:** Porcine fibroblasts were cotransfected with the bi-cistronic vector containing EGFP-T2A-hTBM or hTBM-T2A-EGFP and siRNA targeting EGFP sequence. The expression levels of each gene were analyzed by flow cytometry at 24 h after cotransfection. Summarized bar graphs show means ± SE of three independent replications. **B:** The cell lysates were subjected to western blotting. **C:** After DNase treatment for the removal of residual plasmid DNA, the RNA extracts were subjected to RT-PCR analysis for EGFP. NTC, non-treated cells; siLuc, control siRNA-treated cells; siGFP, target siRNA-treated cells. Two micrograms of each plasmid were used in this figure. These results are representative of three independent experiments.

To examine the effects of RNA interference on the bi-cistronic T2A-Ex constructs, we introduced each bi-cistronic vector (EGFP–T2A–hTBM or hTBM–T2A–EGFP) into porcine fibroblasts simultaneously with EGFP-targeted siRNA (siGFP). Flow cytometric analysis showed that the EGFP signals from both constructs were significantly decreased by siGFP, indicating that the siRNA efficiently downregulated the expression of the target gene. The expression levels of either the upstream- or downstream-positioned hTBM gene were also decreased (Figure 6A). Western blotting with specific antibodies confirmed that the expression of both proteins was markedly decreased (Figure 6B). In addition, we assessed the mRNA levels of EGPF using RT-PCR and found that both transcripts were significantly reduced by siGFP (Figure 6C). These results demonstrate that an siRNA targeting a single gene within the multigene transcript can also affect the expression of the other genes.

## Discussion

The crossing of different transgenic pigs has long been considered the simplest approach to generate multiple transgenic pigs. Recently, however, a viral gene expression system using a 2A peptide sequence has highlighted a novel, practical method to establish multiple transgenic animals. In the context of poly-cistronic expression systems, 2A peptides have been successfully applied in several research fields, including the generation of induced pluripotent stem (iPS) cells from somatic cells [26]–[28], the generation of therapeutic antibody-producing cells [29], and the correction of multigene deficiencies [17]. In addition, generations of multiple transgenic animals using 2A peptides have been previously reported [30]–[33]. However, nobody tried to investigate expression efficiency of the each gene in multi-gene expression system depending on the position.

In terms of generating multiple transgenic pigs, double-transgenic pigs produced by the crossing of hDAF x hCD59 transgenic lines produced heterogeneous gene expression [34]. In triple-transgenic pigs produced by the serial integration of each gene, the expression levels of the transgenes were significantly variable between different tissues, and furthermore, some were not detectably expressed in certain tissues [4]. These observations might be attributed to the different integration loci of each transgene or differences in the promoter control of the genes. The 2A peptide-dependent gene expression system represents a viable alternative to solve this problem. 2A peptide-based multiple transgenic pigs have previously exhibited optimal co-expression of four fluorescent proteins in various tissues [33], indicating that poly-cistronic T2A-Ex system represents an attractive tool for the generation of multiple transgenic pigs, even though the tissue-dependent regulation of promoter activity still must be resolved to ensure that the transgenes are homogenously expressed.

Several reports have suggested that the expression of multiple genes coupled with a 2A peptide is more efficient than IRES-based expression system [31], [35]. Fisicaro et al. reported that a 2A peptide-based tetra-cistronic plasmid did not exhibit any decreased expression of the target genes compared with bi- or tri-cistronic plasmids, at least in vitro [36]. In our experiments using EGFP with different epitope-tagging (HA or Myc), we also identified that the expression levels of the downstream gene were similar to that of upstream gene in the T2A-Ex constructs. However, to be a prospective tool for establishing multiple transgenic animals, the expression patterns of target genes exhibiting different behaviors, including subcellular localization, together with the positional effect of the genes and promoter usage, should be evaluated using the T2A-Ex vector.

Our study using several T2A-Ex constructs revealed that the downstream gene expression likely depends on the expression efficiency of the upstream gene. As demonstrated in our experiment using the Gene A–T2A–EGFP constructs, the protein amount of each downstream EGFP gene was measured by MFI, and it correlated with the expression level of the upstream genes. Because the MFI of the downstream EGFP was significantly correlated with its mRNA expression level, which reflected the relative expression level of the upstream gene, the expression levels of downstream gene appeared to be dependent on the transcriptional efficiency of upstream gene. We next confirmed the influence of the expression efficiency of the upstream gene on the expression efficiency of the downstream gene. The downstream-positioned (HA)HO1 and hCD46 genes were expressed in an upstream gene-dependent manner. Because EGFP was highly expressed compared with hTBM, downstream (HA)HO1 and hCD46 combined with upstream EGFP were also highly expressed compared with the hTBM combination. When the hCD46 gene was positioned upstream of the T2A-Ex construct, the protein expression was barely detected, similar to the expression exhibited by the mono-cistronic vector containing hCD46 alone. Interestingly, the expression of the downstream EGFP and hTBM combined with hCD46 was dramatically decreased. Next, we exchanged the CMV promoter with the CAG promoter to evaluate the extent of promoter dependency on hCD46 expression. The CAG promoter was sufficient to induce hCD46 expression, and the expression of the upstream hCD46 was accompanied by the expression of the downstream hTBM.

Because proteins targeted for different subcellular compartments can be synthesized by different machinery, the differential subcellular targeting might influence the expression of the multiple genes expressed from the same T2A-Ex construct. However, our experiment with the combination of cytosolic EGFP and plasma membrane-targeted hTBM indicated that the two genes were efficiently expressed, regardless of targeted localization. Rather, their expression was affected by the transcriptional efficiency of the upstream gene. Moreover, especially in flow cytometric analysis, the range of difference for EGFP was more extensive than that for hTBM between the groups and it suggests that the degree of antibody binding affinity may attenuate the range of difference between the groups. Accordingly, the fluorescence intensity of EGFP appears to be able to distinguish the change of protein expression levels exquisitely. For this reason, EGFP was used as a downstream gene for comparing the expression efficiency of upstream genes effectively regardless of interference of different antibody binding affinities. Alternatively, immunoblot analyses performed in parallel with flow cytometric analysis precisely reflect the expression change of EGFP and hTBM depending on the position of the genes in T2A-Ex system.

Recently, Kim et al. evaluated the cleavage efficiency of four different 2A peptides and showed that the subcellular localization of the protein was influenced by cleavage efficiency [16]. In our study, we detected small amounts of uncleaved peptide when the EGFP gene was positioned within the upstream region. Nevertheless, the uncleaved form was not detected when the gene encoding the transmembrane protein was located within downstream region (Figure 2C, 3B, 3F, and 5B). These data suggested that the minimal uncleaved form might be cleared by the furin cleavage system within the ER [37] or by signal peptide cleavage of the type I transmembrane protein [38]. Consistent with this notion, flow cytometric analysis showed that the transmembrane proteins were efficiently expressed on the cell surface in all cases. In addition, fluorescent microscopy also showed that EGFP positioned either upstream or downstream of the T2A peptide was expressed efficiently in the cytosol, regardless of the targeted localization of the other genes. Consistent with our results, previous studies have shown that an uncleaved form was readily observed using the same EGFP-containing constructs [15], [39], [40]. This result might be attributed to the expression of a large number of mRNA when the EGFP gene is positioned within the upstream region. To clarify this issue, however, diverse gene combinations must be evaluated in various cell types.

RNA interference represents a useful mechanism that produces various cellular phenotypes with limited gene numbers by targeting specific genes to inhibit their expression in cellular- or tissue-specific contexts. Using in situ miRNA regulation, Brown et al. [41] induced the systemic transduction of lentiviral vectors encoding for transgenes with target sequences of endogenous miRNAs and demonstrated the possibility of using miRNAs in segregating transgene expression between different tissues. Endogenous miRNAs commonly recognize the seed region of 3′-untranslated regions (3′-UTRs) of mRNAs [42]. In a recent study, however, Tay et al. [43] demonstrated that endogenous miRNAs can also react with the coding regions of target genes. To examine the effects of endogenously occurring miRNAs on the T2A-Ex constructs, we used siRNAs that targeted the coding sequence of one transgene. The siRNA significantly downregulated the expression of both the specific target gene and the other gene in the T2A-Ex construct, regardless of their position within the vector. Because both genes are transcribed as one mRNA transcript driven by a single promoter, the overall expression of the genes contained within the T2A-Ex construct was expected to be regulated by the single gene-targeted siRNA. Therefore, RNA interference should be considered another factor for transgene selection in a poly-cistronic expression system, such as T2A-Ex system.

In this study, we tested several combinations of genes in the bi-cistronic T2A-Ex vector in porcine fibroblasts and demonstrated that T2A-Ex is a useful system for efficiently expressing multiple target genes, which likely depends on the adequate expression level of the upstream gene. We also found that the target genes were expressed efficiently, even if they were targeted to different subcellular regions. Therefore, the T2A-Ex system is a promising tool for generating transgenic pigs that express multiple target genes for xenotransplantation.

## Supporting Information

Table S1 **The primer sets used for plasmid construction and RT-PCR.**
(DOCX)
