# Supplementary material for: Expression Analysis of Combinatorial Genes Using a Bi-Cistronic T2A Expression System in Porcine Fibroblasts
Source: PLoS One. 2013 Jul 29;8(7):e70486. doi: 10.1371/journal.pone.0070486 (PMC3726604; doi:10.1371/journal.pone.0070486)
Supplement: Table S1 — The primer sets used for plasmid construction and RT-PCR. (DOCX) [file pone.0070486.s001.docx]

**Supporting Information**

**Table S1. The primer sets used for plasmid construction and RT-PCR.**

| **Purpose** | **Primer name** | **Primer sequence (5’-3’)** |
| --- | --- | --- |
| **Plasmid construction** | **EGFP forward (*Nhe* I)** | **AAGCTAGCACCATGGTGAGCAAGGGCGAGG** |
|  | **EGFP reverse (*Xho* I)** | **ATCTCGAGTTACTTGTACAGCTCGTCC** |
|  | **EGFP forward (*Bam*H I)** | **AAGGATCCATGGTGAGCAAGGGCGAGG** |
|  | **EGFP reverse (*Not* I)** | **AAATTGCGGCCGCTTACTTGTACAGCTCGTCC** |
|  | **(HA)HO1 forward (*Nhe* I)** | **AAGCTAGCACCATGTACCCATACGATGTTCCAGATTACGCTATGGAGCGTCCGCAACCCG** |
|  | **hHO1 reverse (*Xho* I)** | **ATCTCGAGTCACATGGCATAAAGCCC** |
|  | **HA forward (*Bam*H I)** | **AAGGATCCATGTACCCATACGATGTTC** |
|  | **hHO1 reverse (*Not* I)** | **AAATTGCGGCCGCTCACATGGCATAAAGCCC** |
|  | **EGFP forward (*Kpn* I)** | **AAGGTACCATGGTGAGCAAGGGCGAGG** |
|  | **HA reverse (Δstop, *Eco*R I)** | **TTGAATTCAGCGTAATCTGGAACATCG** |
|  | **EGFP forward (*Hin*d III)** | **ATAAGCTTATGGTGAGCAAGGGCGAGG** |
|  | **(HA)EGFP reverse (*Xho* I)** | **AACTCGAGTTAAGCGTAATCTGGAACATCGTATGGGTACTTGTACAGCTCGTCCATGC** |
|  | **Myc reverse (Δstop, *Eco*R I)** | **TTGAATTCCAGGTCCTCCTCTGAGATC** |
|  | **(Myc)EGFP reverse (*Xho* I)** | **AACTCGAGTTACAGGTCCTCCTCTGAGATCAGCTTCTGCTCCTTGTACAGCTCGTCCATGC** |
|  | **HA forward (*Kpn* I)** | **AAGGTACCATGTACCCATACGATGTTC** |
|  | **hHO1 reverse (Δstop, *Eco*R I)** | **AAGAATTCCATGGCATAAAGCCCTAC** |
|  | **HA forward (*Hin*d III)** | **ATAAGCTTATGTACCCATACGATGTTC** |
|  | **hTBM forward (*Bam*H I)** | **AAGGATCCACCATGCTTGGGGTCCTGGTCC** |
|  | **hTBM reverse (Δstop, *Eco*R I)** | **ATGAATTCGAGTCTCTGCGGCGTCCGC** |
|  | **hTBM forward (*Hin*d III)** | **ATAAGCTTATGCTTGGGGTCCTGGTCC** |
|  | **hTBM reverse (*Xho* I)** | **ATCTCGAGTCAGAGTCTCTGCGGCGTC** |
|  | **hCD46 forward (*Kpn* I)** | **AAGGTACCATGGAGCCTCCCGGCCGCC** |
|  | **hCD46 reverse (Δstop, *Eco*R I)** | **AAGAATTCGAGAGAAGTAAATTTTAC** |
|  | **hCD46 forward (*Hin*d III)** | **ATAAGCTTATGGAGCCTCCCGGCCGCC** |
|  | **hCD46 reverse (*Xho* I)** | **ATCTCGAGTCAGAGAGAAGTAAATTTTAC** |
| **RT-PCR** | **EGFP forward** | **ATGGTGAGCAAGGGCGAGG** |
|  | **EGFP reverse** | **TTACTTGTACAGCTCGTCC** |
|  | **β-actin forward** | **ATCTGGCACCACACCTTCTACAATGAGCTGCG** |
|  | **β-actin reverse** | **CGTCATACTCCTGCTTGCTGATCCACATCTGC** |
